# Phytostabilization of Heavy Metals and Fungal Community Response in Manganese Slag under the Mediation of Soil Amendments and Plants

**DOI:** 10.3390/toxics12050333

**Published:** 2024-04-30

**Authors:** Hao Wang, Hui Liu, Rongkui Su, Yonghua Chen

**Affiliations:** College of Life and Environmental Sciences, Central South University of Forestry and Technology, Changsha 410004, China; whlikuee@163.com (H.W.); lhuihui1999@163.com (H.L.);

**Keywords:** manganese mining areas, phytoremediation, phytostabilization, spent mushroom compost

## Abstract

The addition of soil amendments and plants in heavy metal-contaminated soil can result in a significant impact on physicochemical properties, microbial communities and heavy metal distribution, but the specific mechanisms remain to be explored. In this study, *Koelreuteria paniculata* was used as a test plant, spent mushroom compost (SMC) and attapulgite (ATP) were used as amendments, and manganese slag was used as a substrate. CK (100% slag), M0 (90% slag + 5% SMC + 5% ATP) and M1 (90% slag + 5% SMC + 5% ATP, planting *K. paniculata*) groups were assessed in a pilot-scale experiment to explore their different impacts on phytoremediation. The results indicated that adding the amendments significantly improved the pH of the manganese slag, enhancing and maintaining its fertility and water retention. Adding the amendments and planting *K. paniculata* (M1) significantly reduced the bioavailability and migration of heavy metals (HMs). The loss of Mn, Pb and Zn via runoff decreased by 15.7%, 8.4% and 10.2%, respectively, compared to CK. *K. paniculata* recruited and enriched beneficial fungi, inhibited pathogenic fungi, and a more stable fungal community was built. This significantly improved the soil quality, promoted plant growth and mitigated heavy metal toxicity. In conclusion, this study demonstrated that the addition of SMC-ATP and planting *K. paniculata* showed a good phytostabilization effect in the manganese slag and further revealed the response process of the fungal community in phytoremediation.

## 1. Introduction

The demand for manganese in China’s battery and chemical industries is constantly increasing. However, the majority of the country’s manganese mines are open-pit mines of a low quality, making both mining and processing difficult. This, in turn, has resulted in the production of large amounts of tailings, electrolytic manganese residue and mine waste [1,2,3,4]. The presence of these issues has led to severe vegetation damage in manganese mining areas, exacerbating water and soil erosion and causing further ecological imbalances. HMs and toxic substances contained in the tailings continuously migrate to rivers, arable land and farmland, posing a huge potential threat to local ecosystems and human and animal health [5,6,7,8]. Therefore, the ecological restoration of abandoned mines and heavy metal pollution (HMP) control are urgent issues to be addressed in the sustainable development process. Phytoremediation mainly includes phytoextraction [9], phytovolatilization [10] and phytostabilization technologies [11]. Compared with physical and chemical remediation methods, they exhibit environmentally friendly, economically beneficial and non-secondary pollution advantages in treating HMP in large sites such as tailing areas [12,13]. Phytostabilization focuses on using tolerant woody plants to reduce the solubility and bioavailability of HMs in soil to limit their entry into water and food chains [11]. Many studies have shown that restoring vegetation in barren heavy metal mining areas and large polluted sites can effectively inhibit water and soil erosion and the leaching and diffusion of HMs [12]. 

Manganese (Mn) slag has a high heavy metal content, poor water retention and fertility, and normal plants are difficult to grow on it [14]. Suitable amendments are needed to improve the soil quality and fertility of the slag during phytoremediation and the selection of manganese-tolerant tree species is also important. Organic fertilizers such as SMC, plant residue compost, livestock and poultry manure and sludge are widely used in phytoremediation. These fertilizers are abundant in organic matter, have a long nutrient utilization time and are slowly released, making them environmentally friendly, cost-effective and effective [15,16,17,18,19]. Inorganic minerals such as ATP and calcium carbonate that are high-quality inorganic soil amendments used in phytoremediation have a large surface area and can reduce the bioavailability and solubility of HMs through adsorption, ion exchange, coordination reactions and co-precipitation reactions [20,21]. The use of organic–inorganic composite amendments has been shown to significantly improve soil quality and fertility, reduce the bioavailability and mobility of HMs and enhance plant stress resistance compared to single amendments [22,23]. *Koelreuteria paniculata* (Sapindaceae) is a deep-rooted deciduous tree, which is characterized by salt and drought tolerance, resilience to infertile soil, fast growth and well-developed roots [24]. As a pioneer woody plant in manganese mining areas, it has been proven to have good tolerance to HMs such as Mn, Pb and Zn. With the application of soil amendments, it can survive and exhibit exceptional plant fixation effects in manganese slag and lead–zinc slag [25].

The symbiosis and competition between plants and microorganisms promote their adaptation to heavy metal stress environments, while also strongly affecting the bioavailability and mobility of HMs in soil. Plant roots recruit specific microbial communities and provide beneficial nutrients and energy. Some beneficial bacteria and fungi, playing the role of plant-growth-promoting microorganisms (PGPMs), can indirectly promote plant growth and reduce heavy metal toxicity in plants by inducing the plant’s own defense mechanism, or can directly promote plant growth by contributing mineral elements and secreting specific enzymes [5]. PGPMs can also affect and change the bioavailability and mobility of HMs through bioenrichment, bioleaching and bioexclusion processes [26]. The application of different types of organic amendments causes changes in the soil microbial diversity and community structure to varying degrees and further affects plant growth and stress resistance by influencing soil nutrient cycling and the occurrence states of HMs [16,17,27]. Our laboratory has been conducting research on the phytoremediation of heavy metal-contaminated soil for over 10 years [28,29]. Through screening more than 20 types of heavy metal-tolerant woody plants and extensive research and testing on soil amendments [25,30,31], we essentially clarified the stress resistance, heavy metal tolerance and detoxification mechanisms of woody plants such as *K. paniculata* and *Paulownia fortune* at the molecular level [32,33,34], and found that the application of SMC-ATP composite amendments has a small effect on the bacterial diversity in manganese slag, but significantly changes the fungal diversity. As one of the main decomposers of soil organic matter [35,36], the community structure and species abundance of fungi can directly affect the soil’s nutrient cycle [37] and plant growth [38]. The significant changes in fungal diversity are speculated to play a greater role in improving manganese slag soil quality, promoting plant growth and enhancing the phytoremediation efficiency. The application of different types of organic amendments causes changes in the soil microbial diversity and community structure to varying degrees and further affects plant growth and stress resistance by influencing soil nutrient cycling and the occurrence states of HMs in similar studies [16,17,27]. However, at present, we know very little about the changes in the rhizosphere soil microenvironment, mobilization and transformation laws of HMs, and especially the response mechanisms of fungi in manganese slag under the mediation of applying amendments and plant growth.

In view of this, a pilot-scale experiment was conducted in this study; *K. paniculata* was used as the test plant and SMC-ATP was used as an amendment. The differences in the soil matrix’s physical and chemical properties, the occurrence characteristics of soil HMs and soil fungal community diversity were compared under three groups: manganese slag without plant planting (CK), amended manganese slag without plant planting (M0) and amended manganese slag with *K. paniculata* planting (M1), aiming to explore (1) the effects of amendments on the quality and fertility of manganese slag with and without plants; (2) the specific response mechanism of fungal diversity and community structure changes in manganese slag mediated by amendments and plants in the process of phytostabilization.

## 2. Materials and Methods

### 2.1. Soil Amendments and Plants

*K. paniculata* seedlings were obtained from Liuyang Baija Nursery Stock Base, with a height of approximately 80 cm. SMC, mainly composed of materials such as cottonseed hulls, wheat bran, rice bran and corn cobs, was the substrate after cultivating enoki mushroom, and its general physicochemical properties are shown in Table 1. ATP was obtained from a mining area in Gansu Province, mainly composed of hydrated magnesium aluminum silicate, with a pH of approximately 8.0. The manganese slag was obtained from a tailing pond in a manganese mine in Xiangtan City, Hunan Province, and its general physicochemical properties can be found in Table 2 (CK_b_) and Table 3 (CK_b_).

### 2.2. Pilot-Scale Experiment 

The entire study was conducted in the nursery of Central South University of Forestry and Technology in Changsha City, Hunan Province, from January to December 2022. The area has a typical subtropical humid climate with an average annual temperature of about 17.4 °C, an average annual precipitation of about 1361.6 mm, and an average relative humidity of 82%. A pilot-scale experiment using glass containers was conducted to simulate a manganese tailing area. The container was 100 cm long and wide, with an effective height of 80 cm, and had drainage pipes installed at both the top and bottom to collect runoff and seepage rainwater under simulated rainfall. The manganese slag (90%, mass) and amendments (10%, SMC/ATP = 1, mass) were thoroughly mixed to obtain the amended manganese slag. *K. paniculata* seedlings with good and consistent growth were selected for the study. Two blank control groups were set up: manganese slag (CK) and amended manganese slag without plants (M0). The treatment group was amended manganese slag with six *K. paniculata* trees planted (M1), with three replicates. In January 2022, both the manganese slag and the amended manganese slag were added to each device, respectively, followed by the addition of 30 L of tap water to each one. After being covered with plastic film, they were activated for two months. In early March 2022, all the films were removed and *K. paniculata* trees were planted in M1, while the other two control groups were not treated. Each device only received natural rainwater and weed control was strictly implemented. After the plants had been planted for 6 months, simulated rainfall was carried out according to the method of Ouyang [14]: the water volumes for light rain, moderate rain, heavy rain and torrential rain were 2, 10, 20 and 40 L/12 h, respectively; the flow rate was uniformly controlled at 40 L/h using a flowmeter and light rain, moderate rain, heavy rain and torrential rain were sprayed evenly for 3, 15, 30 and 60 min, respectively, within a 12 h period and three replicates were performed. Rainwater from surface runoff and subsurface seepage was collected during the rainy period. The early stage of the study was characterized by pre-planting, whereas the middle stage was defined as the period following the stabilization of plant growth, approximately 6 months after planting. Similarly, the late stage of the study was defined as the period 9 months post planting. Soil physicochemical properties, occurrence state of HMs and plant growth data were monitored. After the experiment, plants were harvested and soil samples were collected for test and data collection, followed by further analysis. 

### 2.3. Soil and Plant Sample Collection 

Soil samples in the pilot-scale experiment were collected using a five-point sampling method at a depth of 10 cm to 20 cm at the early, middle and late stage of this study. The samples were cleaned of impurities, air-dried, sieved and stored in a refrigerator for soil physicochemical properties and heavy metal content and chemical form analysis. A portion of the soil sample collected at the end of the study was stored in a −80 °C freezer for soil fungal diversity analysis. *K. paniculata* was harvested at the end of the study, rinsed with deionized water, and divided into roots, stems and leaves. The three parts were dried at 105 °C for about 30 min, and then dried at 75 °C to a constant weight, sealed and stored for determination of biomass and heavy metal content.

### 2.4. Analysis of Soil Basic Physicochemical Properties 

Soil pH was measured with a pH meter (INESA, Shanghai, China), with a water-to-soil ratio of 2.5:1. Soil water content and porosity followed the method of Nong et al. [39]. Soil organic matter content (SOM) was measured by oxidation–dilution heat method with potassium dichromate according to Lu et al. [40]. The total and available content of nitrogen, phosphorus and potassium in the soil were measured by the method of Bao [41]: total nitrogen (TN) was determined by sulfuric acid digestion–semi-micro Kjeldahl method; available nitrogen (AN) was measured by diffusion dish method; total phosphorus (TP) was measured by molybdenum antimony colorimetry; available phosphorus was measured by carbonate buffer extraction and molybdenum antimony colorimetry; total potassium (TK) was measured by H_2_SO_4_–H_2_O_2_ digestion flame photometry; available potassium (AK) was measured by NH_4_OAc extraction flame photometry. Soil enzyme activity was measured using the methods of Ma et al. [42]: sucrose (SC) activity was determined by 3,5-dinitrosalicylic acid colorimetry; urease (UE) activity was measured by phenol–sodium hypochlorite colorimetry.

### 2.5. Determination of Heavy Metal Content and Chemical Form in Manganese Slag 

A mass of 0.500 g of screened soil sample was weighed and placed in a triangular bottle. Then, 10 mL of aqua regia was added, and the sample was digested on a 170 °C hotplate until the brown fumes disappeared. After adding 3 mL of perchloric acid, the sample was further digested until it became colorless and transparent. After filtration, the soil solution was adjusted to 50 mL with ultra-pure water for determination of heavy metal content in manganese slag. A modified three-step BCR (bioavailability-based sequential extraction procedure) method was used to analyze the chemical forms of HMs in manganese slag, and different forms of HMs in the soil solution were obtained after using the same method of digestion [43]. Finally, the concentrations of Mn, Pb and Zn in all soil solutions were determined by flame atomic absorption spectrophotometry (FAAS, AA-7002, Thermo Fisher Scientific, Waltham, MA, USA).

### 2.6. Plant Sample Analysis 

The dry weight of each part (roots, stems and leaves) of *K. paniculata* was measured using an electronic balance. After grinding each part of the plant into powder, the samples were digested with nitric acid and perchloric acid and analyzed for Mn, Pb and Zn concentrations by flame atomic absorption spectrophotometry (FAAS, AA-7002, Thermo Fisher Scientific, Waltham, MA, USA) [33]. 

### 2.7. Collection of Soil Fungi DNA and Biological Information Analysis 

Genomic DNA was extracted from all the soil samples using MagPure Soil DNA LQ Kit (Magen Biotechnology Company, Guangzhou, China) per the manufacturer’s instructions. DNA integrity and concentration were measured with NanoDrop 2000 (Thermo Fisher Scientific, Waltham, MA, USA) and agarose gel electrophoresis. Extracted DNA was stored at −20 °C until use. DNA was amplified with Takara Ex Taq (Takara Bio Inc., Dalian, China) using fungal ITS genes and barcoded primers. ITS1 variable regions of ITS genes were amplified with universal primers ITS1F (5′-CTTGGTCATTTAGAGGAAGTAA-3′) and ITS2 (5′-GCTGCGTTCTTCATCGATGC-3′). The amplicon quality was verified using agarose gel electrophoresis. The purified and quantified PCR products were subjected to sequencing on an Illumina NovaSeq 6000 (Illumina Inc., San Diego, CA, USA; OE Biotech Company, Shanghai, China). The representative read of each ASV was selected using the QIIME2 package (2020.11). All representative reads were annotated and blasted against the Unite database using q2-feature-classifier with the default parameters. QIIME2 software was used for alpha and beta diversity analysis. The linear discriminant analysis effect size (LEfSe) and linear discriminant analysis (LDA) method was used to compare the taxonomy abundance spectrum.

### 2.8. Data Analysis and Processing 

The data were recorded and organized using Microsoft Office Excel 2021. Soil physicochemical properties, heavy metal content and microbial alpha diversity indices were analyzed using Duncan’s multiple range test in single-factor ANOVA of IBM SPSS (version 27.0). If the difference was significant, it was marked as *p* < 0.05. All data were expressed as the mean and standard deviation (S.D.) of three replicates (*n* = 3). Pearson’s linear correlation analysis was used to study the relationship between environmental factors and microbial abundance, and two significance levels (0.05 and 0.01) were used to indicate the results. Principal coordinate analysis (PCoA) based on fungal ASV Bray–Curtis distance was used to study the differences in fungal community structure. Redundancy analysis [44] was used to investigate the correlation between environmental factors and fungal community structure. The Mantel test was utilized to detect the correlation between fungal ASV Bray–Curtis distance and environmental factors in R (version 4.1.3) using the ggplot2 package (version 3.3.3) and linkET package (version 0.0.7.4), revealing the primary driving force of fungal community structure.

## 3. Results

### 3.1. Differences in Substrate Physicochemical Properties and Soil Enzyme Activity among Different Groups

From the differences in the physicochemical properties of the three substrates at the early and late stage of this study (Table 2 and Table 3), it can be seen that the application of the amendments at the early stage significantly increased the pH of the manganese slag from 7.48 to 7.67, and the moisture content and total porosity also significantly increased by 8% and 9%, respectively (*p* < 0.05). Meanwhile, the TN, TP and SOM in the amended manganese slag (M0 and M1) were significantly higher than those in the manganese slag (CK) (*p* < 0.05), with increases of 194%, 41% and 2%, respectively. In addition, the content of AN, AP and AK in the amended manganese slag also increased significantly, by 175%, 210% and 15%, respectively. This indicates that the application of the amendments optimized the soil quality and fertility of the manganese slag and the water retention capacity was also enhanced. 

At the late stage, the pH of the substrate in each group showed a trend of M1 > M0 > CK (*p* < 0.05), and compared with early stage, the pH of the substrate in CK decreased slightly, while in M0 it decreased significantly (*p* < 0.05), and in M1 it increased significantly (*p* < 0.05). At the same time, the moisture content of the substrate in each group showed a significant decrease compared with the early stage (*p* < 0.05), while the total porosity increased significantly (*p* < 0.05), and the moisture content and total porosity of the substrate in each group at the late stage showed a trend of M1 > M0 > CK (*p* < 0.05). Moreover, there was no significant change in the TN, TP and SOM in CK at the late stage (*p* > 0.05), but the TK decreased significantly (*p* < 0.05). And the TN, TP, TK, AP, AN and SOM in the amended slag (M0 and M1) all significantly decreased (*p* < 0.05), but were still much higher than those in CK. It can be seen that throughout the entire experimental period, the soil fertility and water retention of the amended slag were significantly better than those of the manganese slag, and the growth of *K. paniculata* significantly increased the pH of the amended slag.

**Figure 1:**
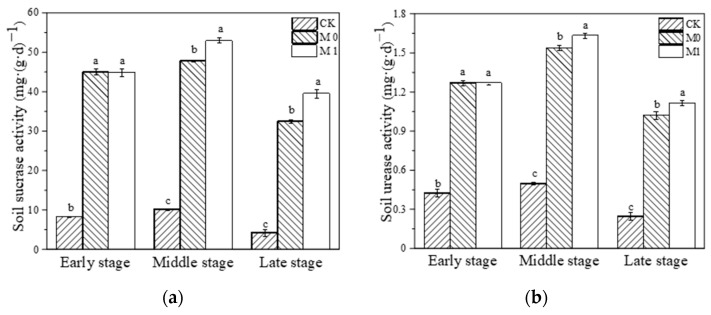
Soil sucrase activity (**a**) and soil urease activity (**b**) at different stages. Different lowercase letters represent significant difference among different groups at the same stage (*p* < 0.05).

During the whole study, we dynamically monitored the SC and UE activity in each substrate. We found that the activity of these two enzymes showed a trend of initially increasing and then decreasing in all three groups (Figure 1). The application of the amendment significantly increased the SC and UE activity in the amended manganese slag (M0, M1) at the early stage compared with CK (*p* < 0.01). And at the middle and late stage of this study, the activity of the two enzymes in M0 and M1 was significantly higher than that in CK during the same period, with a trend of M1 > M0 > CK (*p* < 0.05). It is evident that the application of SMC-ATP significantly increased the SC and UE activity in the manganese slag during this study and maintained it at a higher level, while the growth of *K. paniculata* effectively enhanced the activity of these two enzymes in the amended manganese slag.

### 3.2. The Phytostabilization Effect of K. paniculata

According to the difference in the heavy metal content at the early and late stage in each substrate (Table 4), the content of Mn, Pb and Zn in all three groups showed a significant decrease to varying degrees at the late stage (*p* < 0.05), with CK > M0 > M1. Among them, the reduction rate of Mn in CK, M0 and M1 was 24.5%, 19.8% and 8.8%, respectively. The reduction rates of Pb were 21.9%, 21.3% and 13.5%, respectively. And the reduction rates of Zn were 25.7%, 22.9% and 15.5%, respectively. The contents of Mn, Pb and Zn in various organs (roots, stems and leaves) of *K. paniculata* were not high, and far from reaching the accumulation level in hyperaccumulated plants (Table 5).

**Figure 2:**
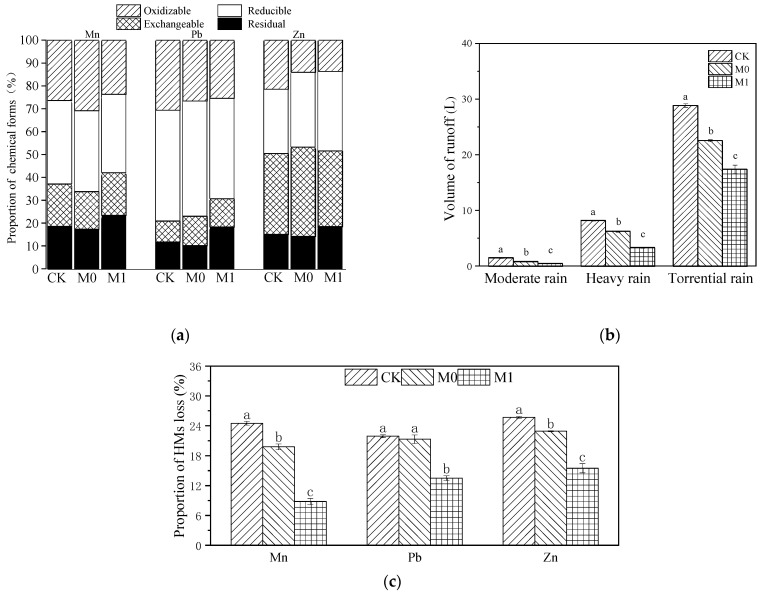
Chemical forms of soil HMs (Mn, Pb and Zn) in different groups after phytoremediation using BCR method (**a**); total volume of surface runoff and subsurface flow in simulated rainfall experiments of different intensities (**b**), different lowercase letters represent significant difference among different groups under the same rainfall intensity (*p* < 0.05); ratio of the total amount of HM (Mn, Pb and Zn) loss with runoff after phytoremediation of different groups to the total amount before phytoremediation (**c**), different lowercase letters represent significant difference among different groups of the same heavy metal (*p* < 0.05).

Due to the significant difference in the initial concentrations of various HMs in manganese slag (CK) and amended slag (M0, M1), we calculated the total amount of Mn, Pb and Zn in each group, as well as the amount of these three HMs lost via rainwater runoff, absorbed by plants and retained in the substrate at the late stage. Then, we performed normalization to obtain the distribution and flow of the HMs in each group (Table 6 and Figure 2c). The runoff loss of Mn and Zn showed a significant difference, with the following trend: CK > M0 > M1 (*p* < 0.05). The loss of Pb did not show a significant difference between CK and M0 (*p* > 0.05), but both were significantly higher than the loss in M1 (*p* < 0.05). The loss of Mn, Pb and Zn in M1 decreased by 15.7%, 8.4% and 10.2%, respectively, compared with CK, while the loss of Mn, Pb and Zn in M0 decreased by 4.7%, 0.6% and 2.8%, respectively. It can be seen that *K. paniculata* absorbed very little of these three HMs, and the largest proportion of HMs was lost through rainwater runoff. It can be observed that the majority of the HM losses were caused by rainwater runoff; the application of the SMC-ATP alone had only a minor effect on reducing the loss of HMs. However, the combined use of SMC-ATP and planting *K. paniculata* resulted in a significant reduction in HM losses.

We conducted a BCR of these three HMs in each group at the late stage and found that the application of SMC-ATP alone (M0) had no significant effect on the residual and reducible proportions of Mn, Pb and Zn in the manganese slag (*p* > 0.05), but significantly increased the proportion of exchangeable Pb and Zn (Figure 2a and Table S1). The application of the amendments and planting *K. paniculata* significantly increased the residual proportion of Mn, Pb and Zn in M1 compared with CK, with increases of 4.8%, 6.6% and 3.4%, respectively. 

Under simulated light rainfall conditions (Figure 2b), no runoff was produced. However, the volumes of runoff in each group differed significantly under simulated moderate rain, heavy rain and torrential rain conditions, with a consistent trend of CK > M0 > M1 (*p* < 0.05), and the difference in the runoff volume in each group increased with increasing rainfall. Under moderate rainfall conditions, the runoff volume in each group did not exceed 2 L. Under heavy rainfall, the average runoff volume in CK reached 8.23 L, which was 2.93 L higher than that in M0 and 4.86 L higher than that in M1. Under torrential rain conditions, the average runoff volume in CK reached 28.87 L, which was 6.29 L higher than that in M0 and 11.44 L higher than that in M1.

To further explore the reasons for the difference in heavy metal runoff loss in different groups, we conducted a Pearson’s correlation analysis of the loss of HMs and runoff volume (Figure S1). The results showed that the loss of Mn, Pb and Zn was significantly positively correlated with the volume of rainwater runoff (*p* < 0.05), while Pb had less mobility compared to Mn and Zn.

### 3.3. The Impact of the Fungal Community Diversity on Phytoremediation Efficiency

The original sequence count of the nine soil fungal samples in the three groups was 715,452, and after filtering out the low-quality sequences, a total of 668,565 optimized sequences were obtained, with an average of 74,285 sequences per sample. These optimized sequences were further clustered into 830 operational taxonomic units (ASVs). The α-diversity analysis revealed that the GOODS (genome organization in selected high-density systems) coverage of the samples was all above 99%, indicating a high testing depth that effectively reflected the actual situation of the samples (Table 7). It was found that the observed species and the species diversity (Simpson index and Shannon index) showed significant differences among the three groups, with CK > M0 > M1 (*p* < 0.05). The observed species in M0 was 58% lower than that in CK and 47% lower than that in M1; the Simpson index in M0 was 77% lower than that in CK and 74% lower than that in M1. The Chao1 index represents the abundance of fungal communities, and it was found that there was no significant difference in the fungal species abundance between M1 and CK (*p* > 0.05), but both were significantly higher than that in M0 (*p* < 0.05). Therefore, it can be concluded that the application of the amendment alone (M0) significantly reduced the species abundance and diversity of the manganese slag (*p* < 0.05), while the combination of planting *K. paniculata* and applying SMC-ATP (M1) significantly decreased the fungal species diversity (*p* < 0.05), but to a lesser extent.

**Figure 3:**
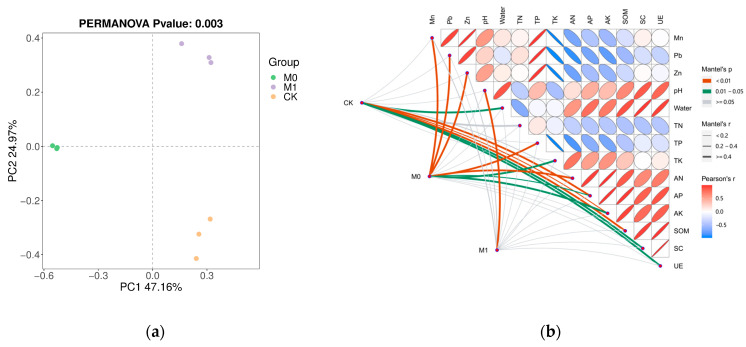
Principle coordinate analysis (PCoA) on ASV level of fungi based on Bray–Curtis distance with PERMANOVA test (**a**); Mantel test (**b**) between environmental factors and fungal community and the pairwise correlation of environmental factors (Pearson’s method). The color of the edge represents Mantel’s *p* and the width of the edge represents Mantel’s r. The pairwise correlation of environmental factors calculated by Pearson’s method was illustrated by the ellipse with colors. The gradient of color represented the r value, *p* value was positively related to the size of ellipse, and the direction of ellipse shows the type of correlation (positive or negative).

Based on the Bray–Curtis distance, the PCoA (principle coordinate analysis) showed significant differences (*p* < 0.05) in the fungal community structure among the three groups (Figure 3a), with the samples within the same group exhibiting good clustering and similarity, while the differences between the different groups were significant. To further understand these significant differences, we conducted a Mantel test between the Bray–Curtis distance based on the ASVs and soil environmental factors, which revealed the main driving factors for the fungal community structure (Figure 3b). In CK, the fungal community structure was strongly correlated (*p* < 0.05) with various factors, including the pH, moisture content, TN, AP, AK, SOM, SC and UE activity. In M0, the fungal community structure was primarily influenced by the content of Mn, Pb and Zn, as well as the TN, TP, TK and their available forms (*p* < 0.05). However, the fungal community structure was only significantly affected by pH in M1 (*p* < 0.05).

**Figure 4:**
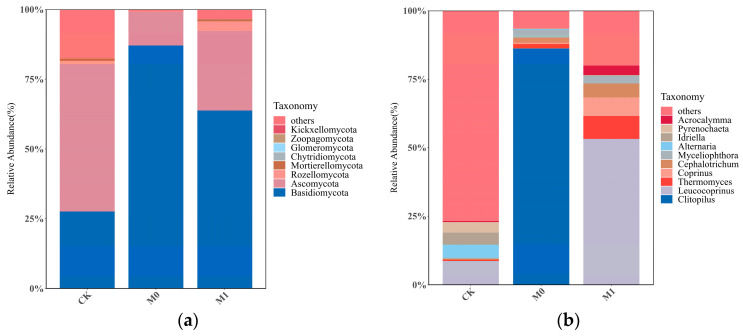
Relative abundances of soil fungi of three groups at phylum level (**a**) and genus level (**b**).

The relative abundance of the top 10 fungal taxa at the phylum level varied significantly among the three groups (Figure 4a). The relative abundance of *Ascomycota* in each sample ranged from 10.04% to 53.48%, while the relative abundance of *Basidiomycota* in each sample ranged from 30.84% to 89.07%. These two phyla were the absolute dominant groups in all the samples and the major fungal taxa in the manganese slag. In CK, the dominant taxa with a relative abundance over 1%, in descending order, were *Ascomycota* and *Basidiomycota*, accounting for 52.83% and 27.58%, respectively. In M0, the dominant taxa with a higher relative abundance, in descending order, were *Basidiomycota* and *Ascomycota*, accounting for 87.03% and 12.06%, respectively. In M1, the dominant taxa with a higher relative abundance, in descending order, were *Basidiomycota*, *Ascomycota* and *Rozellomycota*, accounting for 63.76%, 28.45% and 3.60%, respectively.

The relative abundance of the top 10 fungal taxa at the genus level also significantly differed among the three groups (Figure 4b). In CK, only three genera with a relative abundance of over 1% were dominant, including the genus *Leucocoprinus* in the phylum *Basidiomycota* (8.02%), and the genera *Alternaria* (4.45%) and *Idriella* (4.10%) in *Ascomycota*. In M0, four dominant genera were found, with *Clitopilus* (86.28%) in the phylum *Basidiomycota* being the most abundant, followed by the genera *Cephalotrichum*, *Thermomyces* and *Myceliophthora* in the phylum *Ascomycota*, each with a relative abundance of less than 3%. In M1, up to six dominant genera were found with a relative abundance of over 1%, including the genus *Leucocoprinus* (53.24%) in the phylum *Basidiomycota*, the genera *Thermomyces* (8.43%) and *Cephalotrichum* (5.02%) in the phylum *Ascomycota*, the genera *Coprinus* (6.80%) and *Acrocalymma* (3.46%) in the phylum *Basidiomycota*, and the genus *Myceliophthora* (2.47%) in the phylum *Ascomycota*. 

**Figure 5:**
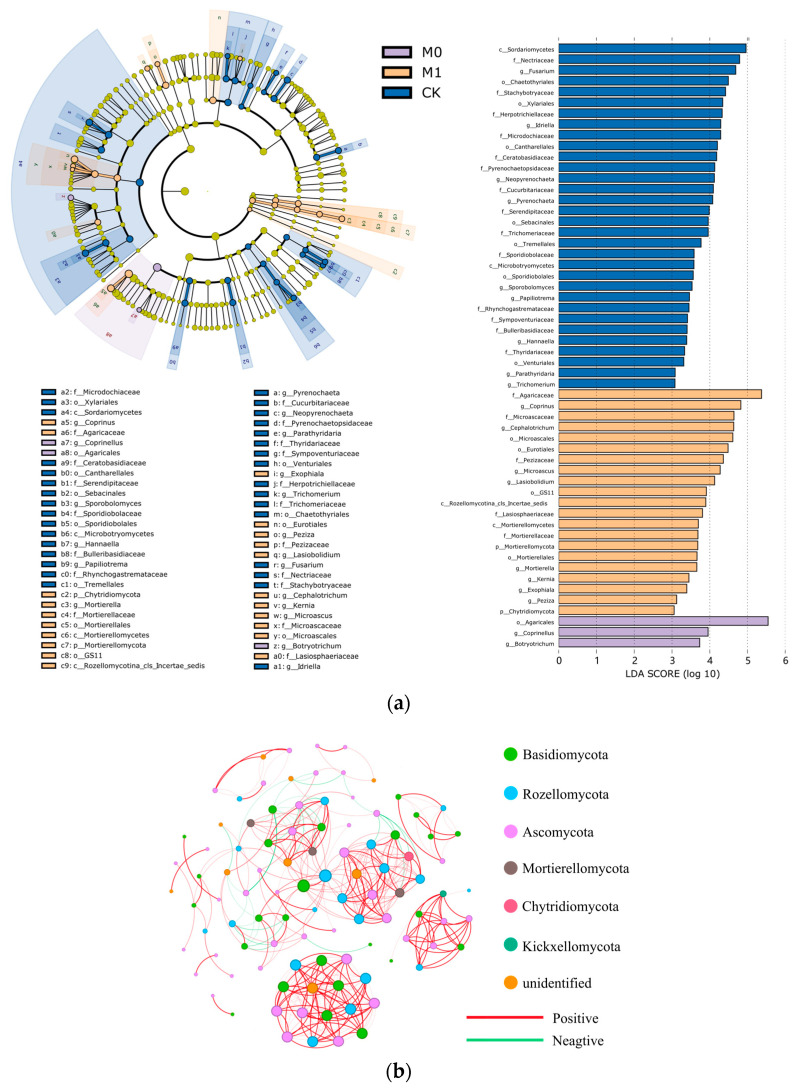
LEfSe and LDA of soil fungal biomarkers (**a**): The layers of circles represent seven levels of taxonomy, from phylum to genus, arranged from the inside out. The colors denote fungal species that are enriched at the corresponding taxonomic level, and the levels of the significant differences. Yellow nodes represent fungal species that do not differ significantly between the groups. Co-occurrence network at the ASV level is shown for six soil samples from M0 and M1 groups (**b**): A connection between nodes indicates a strong correlation (Spearman’s r > 0.7, *p* < 0.01, and ASVs with relative abundance less than 0.5% filtered out). Nodes represent independent ASVs, colored by phylum, and the size of each node is proportional to the number of connections with other ASVs (degree). Edge thickness corresponds to the strength of the correlation between two nodes. A red edge indicates a positive interaction, while a green edge represents a negative interaction.

The LEfSe and LDA analyses revealed significant differences in the fungal community structure and the degree of fungal enrichment among the three groups (Figure 5a and Table S2). In CK, the class *Sordariomycetes* in the phylum *Ascomycota* and the class *Microbotryomycetes* in the phylum *Basidiomycota* were significantly enriched. In M0, only the order *Agaricales* in the phylum *Basidiomycota* was significantly enriched. In M1, in addition to the significant enrichment of the phyla *Basidiomycota* and *Ascomycota*, the phyla *Mortierellomycota*, *Rozellomycota* and *Chytridiomycota* also showed a significant increase in abundance. The biomarkers also varied significantly among the three groups. At the genus level, CK had the most biomarkers, with nine genera, including the genera *Fusarium*, *Idriella*, *Neopyrenochaeta* and *Pyrenochaeta*, showing a high abundance (LDA score > 3). M1 had eight biomarkers, including the genera *Coprinus*, *Cephalotrichum*, *Microascus* and *Lasiobolidium*, while M0 had the fewest biomarkers, with only two genera, *Coprinellus* and *Botryotrichum*.

Co-occurrence networks of fungi can help us better understand the in-depth interactions and connections among different fungal taxa and identify potential key taxa of soil fungi (Figure 5b and Table S3). Therefore, we conducted a co-occurrence network analysis of the fungal taxa at the genus level for the six samples of the amended manganese slag (M0 and M1). The results showed that the co-occurrence network obtained 372 edges among 99 nodes, with 90.3% being positive edges, reflecting a highly mutualistic relationship among the fungal communities in the amended manganese slag. Additionally, the top 10 ASVs based on the BC value were defined as key species in the network (Table 8), providing a better understanding of the ecological niche and mutualistic relationships of the fungi in the amended manganese slag. It was found that the top 10 key species were mainly from the phyla *Rozellomycota*, *Basidiomycota* and *Ascomycota*. Among them, the order *GS11* in the phylum *Ascomycota* (degree 18, BC value 266.9) and the order *Agaricales* in the phylum *Basidiomycota* (degree 18, BC value 266.9) were the most crucial key species.

**Figure 6:**
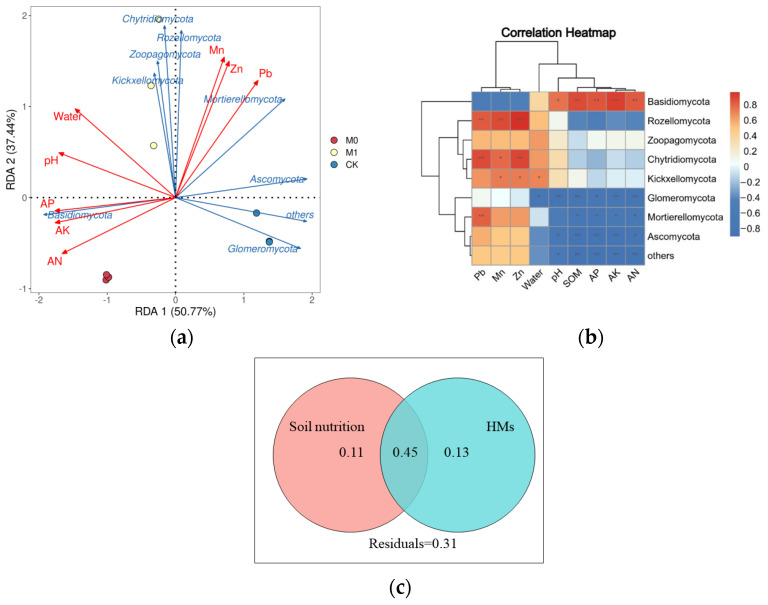
Redundancy analysis (**a**) and correlation analysis by Spearman’s method (**b**) of soil environmental factors and fungal community (top 10 fungal species at phylum level). *, ** and ***, respectively, represent significant differences of (*p* < 0.05), (*p* < 0.01) and (*p* < 0.001); variation partition analysis (VPA) of fungal community (**c**) in all soil samples that can be explained by soil nutrition (SOM, AN, AP and AK) and the content of HMs (Mn, Pb and Zn).

We also conducted redundancy analysis [44] and Spearman’s correlation analysis to explore the relationship between the top 10 fungi at the phylum level and soil environmental factors, which include the HM and nutrient content (Figure 6a,b). The first and second axes of the RDA, which explained 50.77% and 37.44% of the variation, respectively, showed a significant negative correlation between AK and the phyla *Ascomycota*, *Mortierellomycota* and *Glomeromycota*, and a significant positive correlation with the phylum *Basidiomycota* (*p* < 0.05). The moisture content showed a significant positive correlation with the phylum *Mortierellomycota* and a significant negative correlation with the phylum *Kickxellomycota*. The Mn, Pb and Zn content showed a significant positive correlation with the phyla *Rozellomycota* and *Chytridiomycota* (*p* < 0.05). The variation partitioning analysis (VPA) quantified the contributions of the soil nutrient and heavy metal content to the fungal community structure (Figure 6c). These variables explained 45% of the total variation in fungal community structure, with the soil nutrients and heavy metal content explaining 11% and 13%, respectively, while 31% of the variation was left unexplained.

## 4. Discussion

### 4.1. The Effects of Applying Amendments and Planting K. paniculata on the Quality and Fertility of Manganese Slag

The soil quality encompasses its physical, chemical and biological properties, which are crucial in regulating the nutrient and substance flow and transformation, energy metabolism and plant productivity [45,46,47]. Improving the soil quality is a vital part of ecological restoration and is the basis for plant community survival and continual renewal [46]. High concentrations of HMs such as Mn, Pb and Zn in manganese slag are the primary factors limiting plant survival and growth [48]. In addition, the high bulk density and low porosity of manganese slag make it prone to compaction and poor water and nutrient retention, resulting in the loss of moisture, organic matter and nutrients such as nitrogen, phosphorus and potassium [45]. Although woody plants like *K. paniculata* and *P. fortunei* have been found to show good tolerance to HMs such as Mn, Pb, Zn, Cu and Cd and can survive in manganese slag, their growth can be greatly limited due to the lack of moisture and nutrients, resulting in less than ideal phytoremediation effects [25,29].

Adding amendments improved the soil quality of manganese slag before phytoremediation, decreasing its acidity and increasing its fertility and moisture content, providing a suitable soil environment for *K. paniculata* growth and early phytocolonization. Soil with a bulk density of 1.1 to 1.4 g/cm^3^ and a porosity of around 50% is often more ventilated, permeable and capable of retaining moisture, making it more suitable for plant growth [49,50]. In this study, the bulk density and porosity of the manganese slag were 1.62 g/cm^3^ and 38.80%, respectively, with a low moisture content of only 24.32% and a low SOM, TN, TP, AN, AP and AK content, which were not conducive to plant growth. However, the bulk density of the amended manganese slag (M0 and M1) decreased to 1.39 g/cm^3^ and the porosity increased to 48% with the application of SMC-ATP, resulting in a looser and wetter texture. This is consistent with the research of Chen et al. [27]. The application of SMC not only increased the SOM, TP and TN in the soil but also significantly increased the AN, AP and AK, which is consistent with earlier research conclusions, indicating that the SMC played a crucial role in increasing soil nutrient storage and improving nutrient circulation, thereby greatly enhancing the soil’s storage potential for carbon and nitrogen [51,52]. The large number of nanoscale channels present in ATP give it some unique physicochemical characteristics, making it widely used as a soil amendment to improve nutrient utilization, regulate soil bulk density and porosity, and enhance the soil moisture retention capacity, etc. [20]. The application of ATP also resulted in a significant increase in the pH of manganese slag. Adding ATP during composting has been shown to significantly increase the pH of the substrate, similarly [18]. This is because ATP itself is alkaline, neutralizing some H^+^ in the manganese slag. Additionally, during composting, ATP promoted ammonification, nitrification reactions and the release of NH^3^, which are the main reasons for the increase in substrate pH after composting [53].

The growth and development of plants depend on a long-term stable and suitable soil environment, particularly in regard to the soil pH, moisture, total amount and available forms of soil nutrients such as nitrogen, phosphorus and potassium, which directly affect soil productivity [39,54]. The pH of the amended manganese slag was increased after substrate maturation, but the changes in the pH of each group at the late stage were different, and plant growth, microbial action and rainfall may be the cause of these differences. The moisture content and porosity of each group at the late stage were ranked as M1 > M0 > CK, with the moisture content of M1 being almost twice that of CK (13.69%) and the porosity of M0 (50.79%) and M1 (52.21%) maintaining an appropriate value of around 50%. The moisture content, TN, TP and AN, AP and AK in M0 and M1 were significantly decreased at the late stage, but significantly higher than in CK. Soil enzymes produced by microorganisms and plant roots are vital driving factors for substance circulation and energy flow in soil. Almost all biological and chemical processes in soil are highly dependent on enzyme catalysis, so soil enzyme activity can dynamically reflect changes in the quality and fertility of soil [55]. SC catalyzes the hydrolysis of sucrose into glucose and fructose, providing nutrients for microorganisms and plants [56]. UE catalyzes the hydrolysis of urea to produce ammonia and carbon dioxide, which is the key driver of nitrogen utilization, transformation and circulation in ecosystems [44]. HMP has a strong inhibitory effect on soil SC and UE, and their activity can indicate the degree of HMP in soil and the soil health [57], which is why the activity of these two enzymes is low in manganese slag. Adding the amendments (M0, M1) at the early stage significantly increased the activity of SC and UE in the manganese slag, which was further maintained at a much higher level than that in CK during the entire study. Meanwhile, the growth of *K. paniculata* exhibited the effect of maintaining the activity of these two enzymes better in the amended manganese slag (M1) than when applying the amendments alone (M0). And all these results indicated that adding the amendments significantly enhanced the fertilizer retention and moisture retention capacity of the manganese slag during the entire study, and the growth of *K. paniculata* further consolidated and promoted these changes, which, crucially, has positive significance for enhancing *K. paniculata* tolerance and improving phytoremediation efficiency.

### 4.2. The Ecological Interception Effect of Applying Amendments and Planting K. paniculata on Heavy Metals in Manganese Slag

In this study, *K. paniculata* grew well on the amended manganese slag, with no visible damage to its aboveground tissues. The roots of *K. paniculata* were well developed and densely spread throughout the different soil layers in the experimental devices. In M1, the loss of Mn, Pb and Zn decreased by about 15.7%, 8.4% and 10.2%, respectively, compared with that in CK. In M0, the loss of Mn, Pb and Zn decreased by about 4.7%, 0.6% and 2.8% compared with CK. The pilot-scale experiment system we constructed effectively reduced rainwater runoff and exhibited a good ecological interception effect on HMs such as Mn, Pb and Zn in the manganese slag during the simulated rain events of different intensities. As for the occurrence characteristics of HMs in the system and the results of the simulated rainfall experiments, the vast majority of HM loss from the substrates in each group occurred via rainwater runoff, and the total amount of Mn, Pb and Zn enriched in the tissues of *K. paniculata* was very low, amounting to less than 0.01% of the initial amount in the substrates. Overall, applying the amendments alone only slightly reduced the loss of HMs from the substrates, but the combined application of the amendments and planting *K. paniculata* significantly reduced the loss of HMs. The system we constructed in this study demonstrated a good phytostabilization effect and exhibited excellent ecological interception in inhibiting the leaching of HMs from the manganese slag during rainfall events. Generally, there may be two possible factors that contributed to these results.

Firstly, the ecological interception system established in this study buffered rainwater erosion and effectively controlled rainwater runoff. In areas with sparse vegetation, precipitation directly impacts the unprotected surface, with a small portion infiltrating into the underground, while the majority quickly gathers at the surface soil particles to form surface runoff, flowing towards the surrounding water systems [58]. This vicious cycle will continuously exacerbate soil degradation [59]. Heavy metal mining areas are typical areas of vegetation degradation, with serious soil erosion. Ouyang [14] and Huang [60] also reached similar conclusions in their research on manganese mining areas, where most HMs lost from the soil were carried by rainwater erosion and leaching, diffusing into surrounding farmland, cultivated land and water environments. As such, it is clear that the foremost objective of controlling the migration of HMs towards the surrounding environment is to preserve the water and soil in the mining areas by inhibiting the runoff of rainwater, especially the diffusion of surface runoff. Rainfall on land covered by vegetation is divided into three parts: interception loss, throughfall and stemflow. In their study on the impact of rainfall on *Larrea divaricata*, Magliano et al. found that interception loss, throughfall and stemflow accounted for 9.4%, 78.6% and 12.0% of the total rainfall, respectively, and 21% of the rainfall was effectively reduced in kinetic energy through the role of vegetation, preventing it from directly impacting the surface soil [61]. This indicates that the presence of vegetation can buffer the direct impact of rainfall on the ground to some extent and reduce the generation of runoff. The above research conclusion is similar to the results of our simulated rainfall experiments; under moderate, heavy and storm rainfall conditions, there was a significant difference in the runoff volume among the three groups, with the results showing that M1 < M0 < CK. The correlation analysis also showed a significant positive correlation between the loss of Mn, Pb and Zn and the volume of rainwater runoff. At the late stage, the manganese slag (CK) showed severe soil compaction; the lack of vegetation buffering also resulted in an increase in surface runoff and a decrease in subsurface flow during heavy rainfall. Over time, these consequences further aggravated the heavy metal losses, and reduced rainwater infiltration also caused the soil to become increasingly dry and compacted. On the contrary, the amended manganese slag (M0 and M1) did not show any signs of compaction during this study. Due to its loose soil texture, rainwater was able to quickly infiltrate the soil, thereby reducing surface runoff to a great extent. In addition, the improved moisture holding capacity helped to collect more subsurface flow from the rainwater. As a result, it could maintain its own humid and loose condition while also minimizing the loss of HMs. After the restoration of vegetation in the mining areas, plants are able to control HMP by intercepting, precipitating and filtering runoff [16]. It should be noted that adding the amendments and planting *K. paniculata* (M1) exhibited a significant advantage over using the amendments alone (M0) in terms of heavy metal interception in the manganese slag, showing that *K. paniculata* plays a crucial role in the ecological interception process. From the perspective of runoff alone, the presence of trees (such as *K. paniculata*) can reduce surface runoff. As well as this, their well-developed roots are vital in phytostabilization and the stabilization of soil and water.

Secondly, this ecological interception system effectively reduced the mobility and bioavailability of HMs in the manganese slag. There are complex interactions among plant–microbe–soil HMs, and changes in the soil physicochemical properties, root exudates and microbial activity can greatly affect the circulation and movement of HMs in soil by changing their physical and chemical forms. Generally, the acidification, chelation and protonation of root exudates can lead to the migration of HMs, while the precipitation, alkalization and chelation produced by microbial activity lead to their fixation. Meanwhile, the biogeochemical processes of PGPMs, such as microbial enrichment, leaching and exclusion, can alter the form and mobility of HMs and promote microbial adaptation to the environments enriched with HMs [16]. HMs in soils exist in four chemical forms according to their stability, namely, residual, oxidizable, reducible and exchangeable. The residual and oxidizable forms are relatively stable and weakly migratory, while the exchangeable and reducible forms are more bioavailable and mobile. In this study, heavy metal loss was significantly positively correlated with the volume of rainfall runoff, and significantly negatively correlated with the proportion of residual forms of Mn, Pb and Zn in the substrates (*p* < 0.05). When the amendments were added alone (M0), the proportion of residual forms of HMs did not change significantly compared to those in the manganese slag (CK), and the loss of the three HMs decreased slightly compared to that in CK. However, after adding the amendments and planting *K. paniculata* (M1), the proportion of residual forms of Mn, Pb and Zn in the manganese slag significantly increased compared to CK, and the heavy metal loss also significantly decreased. This is similar to the conclusion of another study [62], that showed that the addition of amendments with tolerant woody plants can effectively reduce the bioavailability and mobility of HMs. Soil pH can also significantly affect the bioavailability and mobility of HMs, and the migration rate of most HMs will decrease with increasing pH [5]. Interestingly, the trend of pH change in the substrate in each group was totally different after phytoremediation. The pH of CK did not change significantly, that of M0 decreased significantly, while that of M1 increased significantly. The application of the amendments reduced surface runoff during rainfall and also enhanced the water holding capacity of the manganese slag. The erosion of weakly acidic rainwater may be the main reason for the decrease in pH in M0, and it is clear that the pH increase in M1 was closely related to the growth of *K. paniculata*. As a result, adding the amendments alone did not significantly affect the chemical form stability of the HMs in the manganese slag, and the decrease in the heavy metal loss in M0 was mainly due to the improvement of soil quality by the amendments. However, adding the amendments and planting *K. paniculata* significantly increased the pH of the manganese slag and promoted the transformation of the HMs into less mobile forms, effectively reducing the heavy metal loss via rainwater runoff. This suggests that the growth of *K. paniculata* is crucial to achieving this positive transformation, rather than just the application of amendments. The growth of *K. paniculata* in manganese slag is a typical phytostabilization process, while the addition of the amendments and planting *K. paniculata* had a significant impact on the fungal community structure in the manganese slag. We speculate that the process of phytostabilization in the amended manganese slag is mainly mediated by *K. paniculata* growth, driven by microbial activity, and guided by processes such as precipitation, alkalization and chelation. However, the specific mechanisms require further discussion and analysis.

### 4.3. Response Mechanisms of Fungal Community in Phytoremediation

Some beneficial bacteria and fungi, as PGPMs, indirectly or directly induce plant defense mechanisms by promoting the secretion of plant root exudates and their own specific enzymes, changing the bioavailability and mobility of nutrients and HMs, and stimulating plant growth, resisting plant pathogens and coping with heavy metal stress, thus reducing the toxicity of HMs to plants [5]. Many studies have shown that the addition of organic fertilizers can significantly alleviate the stress of HMs on plants by increasing the species diversity and richness of fungi and bacteria [16,17]. In contrast to other studies’ results, in our study, the abundance and diversity of fungal species in the untreated manganese slag (CK) were the highest. Adding the amendments alone (M0) led to a sharp decrease in fungal species abundance and diversity, while adding the amendments and planting *K. paniculata* (M1) also significantly reduced the abundance and diversity of fungal species compared to in CK, but the decrease was much smaller than that in M0. Despite this, some of our other research results indicate that the reduction in the fungal abundance and diversity in manganese slag may not necessarily have a negative impact on the phytoremediation process.

Soil HMP can lead to the death of sensitive microorganisms in the short term, while there are efficient microbial communities that can tolerate or transform high concentrations of HMs in tailing areas in the long term. It can rapidly change or even destroy the structure of the original community and form new soil microbial communities [63,64]. Some studies have shown that in environments with low carbon sources, nutrients and drought or heavy metal stress, soil fungi are generally dominated by ascomycetes [65,66], which is consistent with our research results. In our study, a tolerant fungal community structure dominated by *Ascomycota* (relative abundance of 52.83%) was formed in the manganese slag. *Ascomycota* has a strong tolerance to oligotrophic and heavy metal stress, achieving a higher resource utilization efficiency in harsh environments [67]. Due to the long-term exposure to high concentrations of HMs, at the genus level, the dominant species in CK with a relative abundance exceeding 1% were only *Leucocoprinus* (8.02%), *Alternaria* (4.45%) and *Idriella* (4.10%). This indicates that these three genera exhibit strong adaptation to heavy metal stress, with *Alternaria* mostly being a plant pathogenic fungus, while being proven to display strong resistance to HMs and environmental stress [63,65]. After the addition of the amendments alone (M0), significant changes occurred in the microbial community structure of the manganese slag, with the abundance of the phylum *Basidiomycota* increasing to 87.03% and the genus *Clitopilus* becoming the dominant species (86.28%) under this phylum. The relative abundance of the other three dominant genera was only between 1% and 3%. Similar situations have also been observed in related studies, where the addition of organic amendments can significantly increase the abundance of specific microorganisms [66,67]. However, the growth of *K. paniculata* in M1 avoided the sharp decrease in microbial diversity caused by the addition of the amendments alone, and formed a community dominated by *Basidiomycota* (63.76%), with the relative abundance of six fungal genera exceeding 1%, and four of them exceeding 5% in terms of the relative abundance. It can be seen that although the abundance and diversity of the microbial communities in the manganese slag were relatively high, the relative abundance of the dominant genera was generally low, while the addition of the amendments alone led to the rapid proliferation of a single genus, which greatly destroyed the balance of the soil ecology. The growth of *K. paniculata* not only avoided the imbalance of fungal communities in the manganese slag but also increased the number and relative abundance of dominant genera. The Mantel test further verified the above view, where the fungal community in CK was strongly correlated with multiple soil nutrient indicators, and the fungal community underwent long-term natural selection of oligotrophy and heavy metal stress, and was insensitive to HMs while being highly sensitive to nutrient deficiencies. The fungal community in M0 was mainly affected by the content of HMs, TP, TK, AN, AP and AK, while the addition of organic fertilizers brought about nutrient abundance, leading to the overgrowth of a single species but also reducing the adaptation ability of the fungal community to heavy metal stress. However, the structure of the fungal community in M1 was only significantly affected by pH, indicating that the soil quality was improved, nutrients were more abundant, and the adaptation ability of the fungal community to HMs was enhanced, resulting in a more stable community structure.

Plants have the capability to select and recruit specific microbial communities from the surrounding soil, shaping their root microbial communities through a process of highly dynamic bidirectional interactions involving the secretion of plant root exudates, microbial signal molecules and changes in soil physicochemical properties [68,69,70]. While in all of our groups, the phyla *Ascomycota* and *Basidiomycota* dominated the fungal community structure, the addition of the amendments and *K. paniculata* growth (M1) remodeled the fungal community structure at the phylum and genus levels, with *Basidiomycota* replacing *Ascomycota* as the dominant phylum at this level. The selection of microbial communities by *K. paniculata* and the application of SMC are the main reasons for the change in the microbial substrate and fungal nutrient acquisition strategies. According to the r- and k-selection strategies in ecological evolution, microbial communities are generally classified as copiotrophic and oligotrophic, and they are essential for the decomposition of soil organic matter [71]. The fungi in the phylum *Ascomycota* are typical copiotrophic fungi that prefer to decompose and utilize easily degradable carbon sources, whereas those in the phylum *Basidiomycota* are mainly oligotrophic fungi that have strong decomposition abilities for recalcitrant organic matter, such as lignin and cellulose [72,73]. This is highly consistent with the findings of Yang [74] and Li [75] et al., which suggested that the addition of a large amount of lignin and cellulose carbon sources in SMC inhibited the growth of the phylum *Ascomycota*, but greatly increased the abundance of the phylum *Basidiomycota*.

The LEfSe analysis further showed significant differences in biomarkers at the phylum and genus levels among the three groups. The class *Sordariomycetes* of the phylum *Ascomycota* and the class *Microbotryomycetes* of the phylum *Basidiomycota* were significantly enriched in CK, constituting the backbone of the fungal network in the manganese slag, and within it, the genus *Fusarium* is a pathogenic fungus [76] that has been demonstrated to display strong resistance to HMs in multiple studies [77,78]. In M0, only the order *Agaricales* of the phylum *Basidiomycota* was significantly enriched, similarly observed by Chen et al. [27] in their study of SMC, with the high abundance of lignin and cellulose in SMC significantly consolidating its ecological niche [79]. At the phylum level, the phyla *Mortierellomycota*, *Rozellomycota* and *Chytridiomycota* were significantly enriched in M1 in addition to the phyla *Basidiomycota* and *Ascomycota*. Based on the RDA, the abundance of the phyla *Rozellomycota* and *Chytridiomycota* were significantly positively correlated with the Mn, Pb and Zn content (*p* < 0.05), and the phylum *Mortierellomycota* was positively correlated with the Pb content. This suggests that the above three species at the phylum level may exhibit strong resistance to HMs. Many studies have shown that the phylum *Mortierellomycota* has an efficient mineral phosphorus solubilization ability. Similarly to previous research [80,81], AP can promote plant growth after being absorbed by plants, which led to a significant negative correlation between the abundance of the phylum *Mortierellomycota* and the AP in this study. The phylum *Chytridiomycota* has the capability to convert organic nitrogen and phosphorus to inorganic forms utilizing different substrates, while promoting protein, chitin, starch and mineral degradation in the process. VPA showed that the soil nutrients and heavy metal content accounted for 69% of the variation in the fungal community structure, indicating that soil fertility and HMP are the two major factors that significantly influence the fungal community structure in manganese slag. 

The addition of SMC introduces abundant organic matter and nutrients such as nitrogen, phosphorus and potassium, transforming the fungal community structure in manganese slag from a copiotrophic type dominated by the phylum *Ascomycota* to an oligotrophic type dominated by the phylum *Basidiomycota*. The fungal co-occurrence network further supports this view, with the order *Agaricales* in the phylum *Basidiomycota* acting as one of the key nodes in the network, significantly promoting the degradation of organic matter in the SMC that is mainly composed of lignin and cellulose, providing sufficient carbon sources and other nutrients to the fungal community and plants. Although the understanding of the phylum *Rozellomycota* is still limited, this species was significantly enriched in M1, with the order *GS11* being another crucial node in the network. In a study by Selvarajan et al. [82], it was found that the phylum *Rozellomycota* was similarly enriched and significantly influenced the soil fungal community composition under HMP involving Mn, cadmium (Cd) and arsenic (As). Unlike other fungi that can only obtain nutrients via permeation, i.e., the secretion of enzymes to degrade organic matter into nutrients that are then absorbed diffusively, *Rozellomycota* can directly obtain nutrients from the organic matter in the environment through phagocytosis [83]. We suggest that as a crucial node closely related to multiple fungal groups, the special nutrient acquisition mode of *Rozellomycota* may play an important role in nutrient cycling and flow in manganese slag, promoting cooperative symbiosis among fungal communities and enhancing the stability of the community structure. Moreover, we believe that the change in the fungal community structure may be the key to explaining the enhanced nutrient retention ability and significant pH increase in the amended manganese slag. Bio-organic fertilizers like SMC are mainly composed of lignin and cellulose, which, unlike inorganic fertilizers, are difficult to decompose and dispose of via runoff. However, they can be decomposed slowly by the beneficial fungi mentioned above, allowing for slow-release fertilization and retention. The process of organic matter decomposition produces some alkaline substances such as amino and hydroxyl groups that can neutralize soil acidic ions, raising the soil pH. The SMC contained a large amount of difficult-to-degrade lignin and cellulose, which decomposed slowly and underwent continuous ammonification and nitrification reactions, leading to the release of NH_3_ [53]. These factors contributed to the slow alkalization of the soil pH in M1. In summary, the significant differences in all the data between the M0 and M1 groups indicated the crucial role of *K. paniculata* in improving the phytoremediation effect; its well-developed root system penetrated deep into the manganese slag, and the secretion of root exudates and signal molecules inhibited the growth of plant pathogens, promoting the enrichment of beneficial bacteria in the rhizosphere and further improving the soil quality, consolidating the stability of the fungal community. This greatly promoted the material cycling, information exchange and energy flow between the plants, soil and microorganisms and exhibited a good phytostabilization effect.

## 5. Conclusions

This study demonstrated that adding SMC and ATP amendments significantly improved the soil quality and fertility of manganese slag, which was further consolidated by the growth of *K. paniculata*. The application of the amendments and the growth of *K. paniculata* reduced the surface rain runoff erosion of the manganese slag and enhanced its soil and water conservation ability, which effectively controlled HMP. Additionally, the growth of *K. paniculata* significantly changed the structure and diversity of the soil fungal communities. Through the regulation of key species in the fungal network, its roots recruited and enriched beneficial fungi, suppressed plant pathogens, promoted organic matter decomposition and nutrient cycling, and reduced the bioavailability and migration of HMs, which had a positive effect on phytostabilization. A fungal co-occurrence network and LEfSe analysis showed that the fungal community transformed from a copiotrophic type to an oligotrophic type, which greatly promoted the decomposition of cellulose and lignin in the SMC; a Mantel test further confirmed that the stability of the fungal community was significantly improved, which directly led to the increase in pH and the maintenance and gradual release of fertilizer in the amended manganese slag, and also promoted the transformation of the HMs into low-migration and low-bioavailability forms. Therefore, the application of amendments and *K. paniculata* in phytoremediation should be encouraged and developed, but further research is needed to deepen our understanding of the response mechanism among plants, HMs and microorganisms.

## Figures and Tables

**Table 1:** Basic physicochemical properties of SMC (mean ± S.D., *n* = 3).

pH	OM (%)	TN (g/kg)	TP (g/kg)	TK (g/kg)	Mn (mg/kg)	Pb (mg/kg)	Zn (mg/kg)
7.23 ± 0.01	52.68 ± 0.52	6.22 ± 0.02	3.13 ± 0.02	8.22 ± 0.02	517 ± 5.03	28 ± 2.00	177 ± 4.51

OM, organic matter; TN, total nitrogen; TP, total phosphorus; TK, total potassium.

**Table 2:** Physicochemical properties of substrates in the three groups before and after phytoremediation (mean ± S.D., *n* = 3).

Group	pH	Moisture Content (%)	Volume Weight (g/cm^3^)	Total Porosity (%)	SOM (%)
CK_b_	7.48 ± 0.01a	24.32 ± 0.19a	1.62 ± 0.02a	38.80 ± 0.81b	2.05 ± 0.04a
CK_a_	7.45 ± 0.01a	13.69 ± 0.40b	1.50 ± 0.02b	43.45 ± 0.68a	2.00 ± 0.03a
M0_b_	7.67 ± 0.01a	32.55 ± 0.27a	1.39 ± 0.03a	47.68 ± 1.27b	4.46 ± 0.02a
M0_a_	7.54 ± 0.02b	22.80 ± 0.45b	1.30 ± 0.02b	50.79 ± 0.49a	4.18 ± 0.04b
M1_b_	7.67 ± 0.01b	32.72 ± 0.15a	1.30 ± 0.03a	47.90 ± 1.12b	4.46 ± 0.03a
M1_a_	7.72 ± 0.02a	26.91 ± 0.36b	1.26 ± 0.01b	52.21 ± 0.20a	3.85 ± 0.03b

CK, manganese slag without any treatment; M0, amended manganese slag without plants; M1, amended manganese slag with *K. paniculata* trees planted; b, before phytoremediation; a, after phytoremediation; SOM, soil organic matter; different lowercase letters in a column represent significant difference in the same groups (*p* < 0.05).

**Table 3:** Nutrient content of substrates in the three groups before and after phytoremediation (mean ± S.D., *n* = 3).

Group	TN (g/kg)	TP (g/kg)	TK (g/kg)	AN (mg/kg)	AP (mg/kg)	AK (mg/kg)
CK_b_	0.16 ± 0.002a	0.33 ± 0.006a	10.16 ± 0.111a	104 ± 4.42a	43 ± 2.00a	335 ± 3.04a
CK_a_	0.15 ± 0.002a	0.32 ± 0.005a	8.24 ± 0.095b	47 ± 2.87b	22 ± 1.07b	283 ± 5.43b
M0_b_	0.47 ± 0.004a	0.45 ± 0.004a	9.79 ± 0.015a	286 ± 2.44a	134 ± 1.10a	385 ± 4.52b
M0_a_	0.40 ± 0.003b	0.23 ± 0.004b	8.74 ± 0.066b	136 ± 2.34b	54 ± 3.40b	441 ± 9.59a
M1_b_	0.47 ± 0.005a	0.45 ± 0.005a	9.78 ± 0.035a	285 ± 4.33a	135 ± 2.00a	385 ± 3.31a
M1_a_	0.30 ± 0.008b	0.34 ± 0.004b	7.99 ± 0.051b	79 ± 3.48b	42 ± 1.90b	369 ± 3.19b

CK, manganese slag without any treatment; M0, amended manganese slag without plants; M1, amended manganese slag with *K. paniculata* trees planted; b, before phytoremediation; a, after phytoremediation; TN, total nitrogen; TP, total phosphorus; TK, total potassium; AN, available nitrogen; AP, available phosphorus; AK, available potassium; different lowercase letters in a column represent significant difference in the same groups (*p* < 0.05).

**Table 4:** Heavy metal content of substrates in the three groups before and after phytoremediation (mean ± S.D., *n* = 3).

Group	Heavy Metal Content (mg/kg)		
	Mn	Pb	Zn
CK_b_	10496 ± 66.8a	3221 ± 19.9a	4224 ± 19.5a
CK_a_	7923 ± 32.0b	2516 ± 10.4b	3139 ± 13.6b
M0_b_	9124 ± 26.9a	2928 ± 6.6a	3824 ± 20.1a
M0_a_	7321 ± 33.5b	2304 ± 21.9b	2950 ± 15.9b
M1_b_	9135 ± 45.8a	2931 ± 17.6a	3835 ± 6.0a
M1_a_	8332 ± 20.5b	2534 ± 24.1b	3242 ± 39.8b

CK, manganese slag without any treatment; M0, amended manganese slag without plants; M1, amended manganese slag with *K. paniculata* trees planted; b, before phytoremediation; a, after phytoremediation; different lowercase letters in a column represent significant difference in the same groups (*p* < 0.05).

**Table 5:** Heavy metal content in different organs of *K. paniculata* after phytoremediation (mean ± S.D., *n* = 3).

Heavy Metal	Heavy Metal Content (mg/kg)		
	Root	Stem	Leaf
Mn	1054 ± 36.1	564 ± 32.7	1036 ± 15.9
Pb	43 ± 1.7	6 ± 0.3	215 ± 11.9
Zn	238 ± 10.0	118 ± 6.7	76 ± 3.8

**Table 6:** Distribution of heavy metals after phytoremediation (mean ± S.D., *n* = 3).

Heavy Metal	Group	Proportion (%)		
		Substrate	Plant	Runoff
Mn	CK	75.5 ± 0.38c	-	24.5 ± 0.38a
	M0	80.2 ± 0.57b	-	19.8 ± 0.57b
	M1	91.2 ± 0.62a	0.005 ± 0.00	8.8 ± 0.62c
Pb	CK	78.1 ± 0.29b	-	21.9 ± 0.29a
	M0	78.7 ± 0.84b	-	21.3 ± 0.84a
	M1	86.5 ± 0.48a	0.001 ± 0.00	13.5 ± 0.48b
Zn	CK	74.3 ± 0.20c	-	25.7 ± 0.20a
	M0	77.1 ± 0.10b	-	22.9 ± 0.10b
	M1	84.5 ± 0.93a	0.002 ± 0.00	15.5 ± 0.93c

CK, manganese slag without any treatment; M0, amended manganese slag without plants; M1, amended manganese slag with *K. paniculata* trees planted; Substrate, amount of heavy metals remaining in the substrate (%); Plant, amount of heavy metals absorbed by plants (%); Runoff, amount of heavy metals lost via runoff; different lowercase letters in a column represent significant difference (*p* < 0.05).

**Table 7:** Fungal richness and diversity indices of substrates in the three groups (mean ± SE, *n* = 3).

Group	GOODS Coverage	Observed Species	Chao1 Index	Simpson Index	Shannon Index
CK	0.999997 ± 0.00a	146 ± 44a	145 ± 43a	0.94 ± 0.01a	5.16 ± 0.17a
M0	0.999981 ± 0.00a	61 ± 12c	60 ± 12b	0.22 ± 0.06c	1.02 ± 0.27c
M1	0.999992 ± 0.00a	116 ± 23b	115 ± 23ab	0.84 ± 0.00b	3.62 ± 0.07b

CK, manganese slag without any treatment; M0, amended manganese slag without plants; M1, amended manganese slag with *K. paniculata* trees planted; different lowercase letters in a column represent significant difference. (*p* < 0.05).

**Table 8:** Topological properties of major keystone species of substrates in the three groups.

ASV	Phylum	Class	Order	Family	Genus	Degree	BC Value
27	*Rozellomycota*	*Rozellomycotina_cls_Incertae_sedis*	*GS11*	uncultured	uncultured	18	266.9
20	*Basidiomycota*	*Agaricomycetes*	*Agaricales*	uncultured	uncultured	18	266.9
40	*Rozellomycota*	uncultured	uncultured	uncultured	uncultured	5	186.4
17	*Ascomycota*	*Sordariomycetes*	*Sordariales*	*Chaetomiaceae*	*Botryotrichum*	9	118.0
10	*Basidiomycota*	*Tremellomycetes*	*Cystofilobasidiales*	*Mrakiaceae*	*Tausonia*	8	89.3
51	*Ascomycota*	*Sordariomycetes*	*Hypocreales*	*Stachybotryaceae*	*Albifimbria*	5	66.0
1	*Basidiomycota*	*Agaricomycetes*	*Agaricales*	*Entolomataceae*	*Clitopilus*	10	61.1
316	*Rozellomycota*	uncultured	uncultured	uncultured	uncultured	7	45.9
345	*Basidiomycota*	*Agaricomycetes*	uncultured	uncultured	uncultured	7	45.9
32	*Basidiomycota*	*Agaricomycetes*	*Agaricales*	*Agaricaceae*	*Coprinus*	7	44.7

BC value, betweenness centrality value.

## Data Availability

The data referred to in this study are available upon request from the corresponding author.

## Supplementary Materials

The following supporting information can be downloaded at: https://www.mdpi.com/article/10.3390/toxics12050333/s1, Figure S1: Pearson correlation analysis of the loss of heavy metals and runoff volume; *, **, and *** respectively represents significant difference (*p* < 0.05), (*p* < 0.01) and (*p* < 0.001); Table S1: Chemical forms of soil HMs (Mn, Pb, and Zn) in different groups after phytoremediation using BCR method (mean ± S.D., *n* = 3). Table S2. The relative abundance of the top 10 fungal taxa at the phylum level in different groups (mean ± S.D., *n* = 3).; Table S3: The relative abundance of the top 10 fungal taxa at the genus level in different groups (mean ± S.D., *n* = 3).
