# Supplementary material for: Phytostabilization of Heavy Metals and Fungal Community Response in Manganese Slag under the Mediation of Soil Amendments and Plants
Source: Toxics. 2024 Apr 30;12(5):333. doi: 10.3390/toxics12050333 (PMC11125594; doi:10.3390/toxics12050333)
Supplement: Supplementary file 1 [file toxics-12-00333-s001.zip › toxics-2986566-supplementary.pdf]

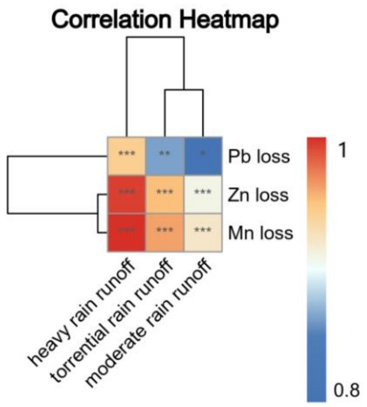

Figure S1. Pearson correlation analysis of the loss of heavy metals and runoff volume; * , ** , and *** respectively represents significant difference ($P < 0.05$)，($P < 0.01$) and ($P < 0.001$).

Table S1. Chemical forms of soil HMs (Mn, Pb, and Zn) in different groups after phytoremediation using BCR method (mean ± S.D., $n = 3$).

Chemical form	Group	Heavy metal (%)		
		Mn	Pb	Zn
Residual	CK	18.66±0.40b	11.80±0.45b	15.16±0.24b
	M0	17.39±0.18c	10.25±0.09c	14.28±0.13c
	M1	23.45±0.15a	18.36±0.47a	18.53±0.23a
Exchangeable	CK	18.49±0.21a	9.21±0.34b	35.35±0.11b
	M0	16.49±0.22b	12.88±0.62a	39.03±0.31a
	M1	18.67±0.31a	12.37±0.39a	33.10±0.20c
Reducible	CK	36.52±0.27a	48.41±0.64b	28.09±0.73c
	M0	35.31±0.33b	50.32±0.06a	32.69±0.60b
	M1	34.23±0.28c	43.91±0.50c	34.76±0.29a
Oxidizable	CK	26.33±0.18b	30.57±0.52a	21.40±0.51a
	M0	30.81±0.46a	26.55±0.54b	14.00±0.53b
	M1	23.65±0.43c	25.36±0.69c	13.61±0.25b

CK, manganese slag without any treatment; M0, amended manganese slag without plant; M1, amended manganese slag with *K. paniculata* trees being planted; different lowercase letters in a column represent significant difference ($P < 0.05$).

Table S2. The relative abundance of the top 10 fungal taxa at the phylum level in different groups (mean ± S.D., $n = 3$).

Taxonomy	Relative abundance (%)		
	CK	M0	M1
Basidiomycota	27.58±0.95	87.03±0.64	63.76±0.69
Ascomycota	52.83±0.26	12.06±0.88	28.45±0.22
Rozellomycota	1.15±0.02	0.21±0.17	3.60±0.12
Mortierellomycota	0.85±0.04	0.01±0.02	0.68±0.20
Chytridiomycota	0.00±0.01	0.00±0.00	0.03±0.02

Glomeromycota	0.02±0.01	0.00±0.00	0.00±0.00
Zoopagomycota	0.00±0.00	0.00±0.00	0.01±0.01
Kickxellomycota	0.00±0.00	0.00±0.00	0.01±0.01
others	17.57±0.74	0.68±0.30	3.46±0.15

CK, manganese slag without any treatment; M0, amended manganese slag without plant; M1, amended manganese slag with *K. paniculata* trees being planted.

Table S3. The relative abundance of the top 10 fungal taxa at the genus level in different groups (mean ± S.D., n = 3).

**Taxonomy**	**Relative abundance (%)**		
	**CK**	**M0**	**M1**
Clitopilus	0.00±0.01	86.28±1.07	0.00±0.00
Leucocoprinus	8.02±2.40	0.07±0.11	53.24±0.42
Coprinus	0.00±0.00	0.46±0.20	6.80±0.18
Acrocalymma	0.36±0.36	0.00±0.01	3.46±0.14
Cephalotrichum	0.33±0.39	1.88±0.26	5.02±0.00
Thermomyces	0.46±0.24	1.55±0.14	8.43±0.84
Myceliophthora	0.40±0.10	2.91±0.74	2.47±0.14
Tausonia	0.01±0.01	0.00±0.00	0.36±0.13
Idriella	4.10±2.64	0.03±0.01	0.31±0.53
Alternaria	4.45±0.15	0.27±0.01	0.15±0.71
others	18.14±3.85	93.45±0.93	80.23±0.22

CK, manganese slag without any treatment; M0, amended manganese slag without plant; M1, amended manganese slag with *K. paniculata* trees being planted.
